# Public Auditing with Privacy Protection in a Multi-User Model of Cloud-Assisted Body Sensor Networks

**DOI:** 10.3390/s17051032

**Published:** 2017-05-05

**Authors:** Song Li, Jie Cui, Hong Zhong, Lu Liu

**Affiliations:** 1 School of Computer Science and Technology, Anhui University, Hefei 230601, China; lisong@ahu.edu.cn (S.L.); zhongh@ahu.edu.cn (H.Z.); 2 Department of Computing and Mathematics, University of Derby, Derby DE22 1GB, UK; L.Liu@derby.ac.uk

**Keywords:** cloud computing, wireless body sensor networks, privacy preserving, public auditing

## Abstract

Wireless Body Sensor Networks (WBSNs) are gaining importance in the era of the Internet of Things (IoT). The modern medical system is a particular area where the WBSN techniques are being increasingly adopted for various fundamental operations. Despite such increasing deployments of WBSNs, issues such as the infancy in the size, capabilities and limited data processing capacities of the sensor devices restrain their adoption in resource-demanding applications. Though providing computing and storage supplements from cloud servers can potentially enrich the capabilities of the WBSNs devices, data security is one of the prevailing issues that affects the reliability of cloud-assisted services. Sensitive applications such as modern medical systems demand assurance of the privacy of the users’ medical records stored in distant cloud servers. Since it is economically impossible to set up private cloud servers for every client, auditing data security managed in the remote servers has necessarily become an integral requirement of WBSNs’ applications relying on public cloud servers. To this end, this paper proposes a novel certificateless public auditing scheme with integrated privacy protection. The multi-user model in our scheme supports groups of users to store and share data, thus exhibiting the potential for WBSNs’ deployments within community environments. Furthermore, our scheme enriches user experiences by offering public verifiability, forward security mechanisms and revocation of illegal group members. Experimental evaluations demonstrate the security effectiveness of our proposed scheme under the Random Oracle Model (ROM) by outperforming existing cloud-assisted WBSN models.

## 1. Introduction

**Figure 1:**
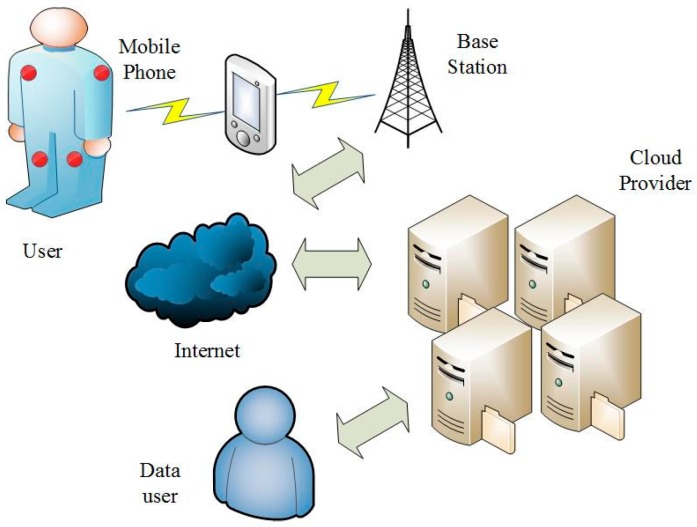
The system model of cloud-assisted WBSNs.

Wireless Sensor Networks (WSNs) have become increasingly popular and find deployments in several IoT applications such as military, transportation, healthcare, etc. Wireless Body Sensor Networks (WBSNs) are an emerging form of WSNs [1], which exploits wearable computing devices for processing applications. Remote healthcare monitoring of patient’s health [2] is one particular example of WBSN deployments, by which doctors can monitor the patient’s health without the need for physically visiting the patients. Given the affordability and easy access [3,4,5] to the sensor and other embedded devices, WBSNs can now be deployed without any major investment costs. In a WBSN deployment scenario, wearable sensor devices collect and send information to distant providers for instant [6] processing of information. Thus WBSNs provide supplements for doctors to initiate instant responses to fatal healthcare conditions, such as sudden infant death syndrome (SIDS). Figure 1 shows the system model of cloud-assisted WBSNs.

A range of deployment architectures for WBSN application models has been proposed in the literature [7,8,9,10]. Deploying wireless protocols such as ZigBee [11] for data transmission in WBSNs applications can enhance the communication services for better user experiences.

In addition to medical applications, WBSNs also find applications in video streaming, data file transfer, 3D video and entertainment applications, including gaming and social networking. A power game-based approach [12] has been proposed to mitigate the communication interference for WBSNs based on social networks.

In order to standardize the specifications of WBSNs, the Institute of Electrical and Electronics Engineers has formulated the IEEE 802.15.6 standard for wireless communication [13]. The aim of this standard is to provide an international standard for low power, short range and reliable wireless communication in the area surrounding the human body. It supports a vast range of data rates for different applications, such as short-range and wireless communications.

One of the prevailing issues in the WBSN technique is the level of security and privacy of the information offered. It is essentially important to ensure data integrity and privacy so that user information can only be accessed by the authorized entities, and further, the stored data used for diagnosis should not be corrupted, etc. However, due to the reality of resource constraints in WBSNs in terms of power, memory, bandwidth capacity, computational resources, etc., ensuring reliability and data security in WBSN applications is still challenging.

IEEE 802.15.6 defines three levels of security: Level 0 is unsecured communication; this is the lowest security level in 802.15.6 where no security measures are conducted. Level 1 is an authentication only scheme, and Level 2 includes both authentication and simultaneous encryption to achieve the highest security goal in 802.15.6. This standard demands that every device participating in the transmission must be ensured with a definite level of security. Furthermore, a pre-shared Master Key (MK) is activated in unicast communication; a Pairwise Temporal Key (PTK) is generated for a single use per session; a Group Temporal Key (GTK) is generated and shared with the corresponding group in multicast communication. Devices participating in the WBSN system must undergo a certain level of security at the MAC layer before exchanging data.

Security research in WBSNs: In general, the communication model of wireless body sensor networks can be divided into two segments. The first segment is the inside-body communication, which denotes the communication between sensors. The second segment is the outside-body communication denoting the communication between the gateway and other network participants such as the service provider, remote monitor and cloud servers.

For processing physiological information of users in WBSNs, various inside-body authentication schemes have been proposed [14,15,16,17,18,19,20,21,22,23,24,25,26,27]. In [14,15], the inter-pulse interval of Electrocardiogram (ECG) and Photoplethysmogram (PPG) have been used to generate cryptographic keys for encryption and authentication. In [16,17], the frequency coefficients of ECG and PPG have been used to generate a cryptographic key. In [18], Jules and Sudan put forward the idea of a fuzzy vault, which is widely used in the field of biometric authentication [19,20,21]. A Physiological Signal Key Agreement scheme (PSKA) based on fuzzy vault has also been proposed [22]. However, the use of extra chaff points in the PSKA scheme increases the computation cost. In [23,24], the modified fuzzy vault method with the ECG signal has been proposed to improve performance. An enhanced fuzzy vault method [25] has also been presented, and the method is applied to the application of key generation based on a fingerprint. In [26], Biel et al. proposed to use the ECG signal for biometric authentication, which needs generating a signal template to verify the identity by a comparative analysis. Due to the use of static templates, this scheme may not achieve good security performance. In [27], a scheme based on time variation ECG feature has been proposed for authentication and to extract the key for authentication/encryption. However, such methods based on physiological parameters lack accuracy, since the signals collected from different parts of the same individual always incur marginal differences. In addition, the physiological signal is time-variant, and so, strict clock synchronization is needed which is difficult to achieve. Furthermore, such schemes face compatibility issues when deployed with different sensor types, thus suffering from practical limitations.

In addition, cryptography technology has been widely used to secure communication in the WBSNs, as well. However, Traditional Public Key Cryptography (TPKC) requires a large number of certificates to be stored and transmitted. Wearable devices used in WBSN suffer from storage and computational limitations; thus, traditional cryptography may not be an effective security solution in resource-constrained WBSN environments. Identity-based cryptography technology, compared with TPKC technology, has the advantage of using a public key for identification and eliminates complex certificate management [28], so it is widely used in a variety of security protocols. However, the existence of the key escrow problem has imposed several challenges for security management. Addressing the key escrow problem, certificate-less cryptography schemes have been proposed [29]. Certificate-less cryptography eliminates both the key escrow problem in identity-based cryptography and certificate management issues in TPKC. In certificate-less cryptography, the key is generally divided into two parts, generated by the user and the Key Generation Centre (KGC), respectively. To this end, certificateless cryptography schemes are more suitable for deployments in resource-constrained WBSN devices.

A few WBSN authentication protocols have been proposed [30,31,32,33,34] based on certificateless cryptography. In [30], Liu et al. propose two certificateless authentication schemes for WBSNs. However, Zhao [31] finds that Liu et al.’s scheme cannot withstand the stolen verifier table attack and proposes an improved scheme to address the verifier table attack. Later, Wang et al. [32] cites that the constant pseudo user identities in Zhao’s scheme [31] are vulnerable for easy identification. Xiong et al. [33] cites that Liu et al.’s scheme [30] lacks scalability and forward security and proposed a new scalable and anonymous authentication scheme for WBSNs. Furthermore, Xiong et al. proposed another revocable and scalable scheme in [34] with the KUNode tree structure (an Identity-based Encryption with Efficient Revocation). Despite the existing methods, WBSNs still need an effective security mechanism for counteracting the resource constraints of wearable devices in applications such as mobile health services (m-health), probability computing, medical social networks, data mining, etc.

In general, people mounted with wearable sensors characterize random mobility as they usually walk around rather than being static [35]. People usually wear medical sensors on their body and use mobile devices such as mobile phones as a gateway for communicating with the remote service provider to access health services.

Based on the concept of m-health, users with the same pathological features may communicate with each other and construct a social network so as to provide richer WBSNs service. In [36], a security framework using probabilistic computation has been proposed. Battery status in mobile devices is crucial in m-health services particularly when users require emergency assistance. In scenarios where the battery status of the mobile devices cannot support long time use, this scheme [36] facilitates the devices by using probability computation to request nearby temporary gateways. However, this scheme includes flaws such as user anonymity and mutual authentication [37]. An improved mobile-healthcare emergency system based on extended chaotic maps has been proposed to enhance the computational efficiency of the WBSNs devices. In [38], a method of health data analysis with privacy protection using paillier homomorphic encryption has been proposed, but the proposed scheme is based on a security assumption that the distributed servers will not collude together, which may not always be true.

Security research in cloud-assisted WBSNs: The emerging cloud computing technology can provide storage and computing supplements to resource-constrained WBSN devices. The cloud-assisted WBSN model has been increasingly researched in the recent past. In cloud-assisted WBSNs, the cloud servers can act as a computational service provider to process the health data collected by WBSNs devices or as a storage service provider to store the health data to build user‘s medical records. In [39], Wan et al. studied a cloud-assisted WBSNs architecture and its applications in pervasive healthcare systems. They focused on the methodologies for transmitting vital sign data to the cloud by energy-efficient routing, cloud resource allocation, semantic interactions and data security mechanisms. In [35], a secure and privacy-protecting key management scheme for cloud-assisted WBSNs in m-healthcare social networks has been proposed. In [40], a multi-valued and ambiguous encryption scheme to ensure data confidentiality has been proposed. In order to ensure the integrity of medical data stored in the cloud, some public auditing schemes for cloud-assisted WBSNs have been proposed [41,42,43]. However in these schemes, the cloud server could obtain user uploaded data directly, and this would result in the disclosure of users’ sensitive medical information.

Processing user’s unprotected sensitive information in public cloud servers certainly includes a potential threat to data security and data integrity. Besides, in some WBSNs applications, such as a community hospital environment, users and doctors in the same community may want to organize a group to share the data stored in cloud servers. Therefore, it is important to construct a public auditing scheme supporting a multi-user model.

Our contributions: Based on the above considerations, this paper presents a public auditing scheme with privacy protection for cloud-assisted WBSNs supporting a multi-user model. The important contributions of this paper include the following:

1. A novel service model supporting a multi-user model in cloud-assisted WBSNs is presented. This service model enhances the user experience of WBSNs services in a community hospital environment.
2. An improved scheme for supporting public auditing and to protect user‘s data privacy simultaneously (under the assumption that the cloud server is not trusted) is implemented. The multi-user model ensures cloud data access only to authorized members from the same group using a secret group key. Furthermore, our mechanism revokes group members exhibiting illegal actions and achieves system forward security by updating the group key. Experimental evaluations prove that our scheme is secure under the Random Oracle Model (ROM) and outperforms existing schemes in cloud-assisted WBSNs applications.

Organization: The rest of the paper is organized as follows: Section 2 presents the preliminaries of our proposal including a bilinear map, elliptic curve cryptosystems, elliptic curve discrete logarithm problem and computational Diffie-Hellman problem along with the security requirements and the system model. Section 3 describes our certificateless public auditing scheme with privacy protection and revocation mechanism in a multi-user model. Section 4 proves the security efficiencies of our scheme under the random oracle model. Section 5 discusses the security features of our scheme, while Section 6 presents the implementation of our scheme with the JPBC (Java Pairing Based Cryptography) cryptographic library and evaluates our model against the existing works. Finally, Section 7 concludes this paper.

## 2. Preliminaries

This section will introduce the mathematical background, the system model and the security requirements for the cloud-assisted WBSN public auditing scheme.

### 2.1. Bilinear Map

Given a cyclic multiplicative group *G* with order *q*; given another multiplicative cyclic group *G_T_* with the same order *q*; A bilinear pairing refers to a map *e*: *G × G → G_T_* that should satisfy the following properties:

1. Bi-linearity: for all *P, Q* ∈ *_R_* *G* and *a, b* ∈ _R_ Z_q_^*^, *e* (*a∙P, b∙Q*) *= e*(*P, Q*)*^ab^*.
2. Non-degeneracy: there exist *P*, *Q* ∈ *_R_* *G* such that *e* (*a∙P*, *b∙Q*) ≠ 1*_GT_*.
3. Computability: for all *P, Q* ∈ *_R_* *G*, there exists an efficient algorithm to compute *e* (*a∙P*, *b∙Q*).

### 2.2. Elliptic Curve Cryptosystem

An Elliptic Curve Cryptosystem (ECC) was independently proposed by Miller [44] and Koblitz [45] in 1985 and 1987, respectively. Compared to RSA (Rivest, Shamir and Adleman algorithm), ECC (Elliptic Curve Cryptography) can achieve the same security requirements with a shorter key-length [46]. Hence, it has recently been widely used in many cryptographic schemes.

An elliptic curve [47,48] is defined over a finite field *F_p_* by equation *E_p_*(*a*,*b*): *y*^2^ = *x*^3^ + *ax* + *b*, where *p* is a large prime and 4*a*^3^ + 27*b*^2^ = 0 mod *p*. The points on this elliptic curve form a cyclic group. Addition in this group is defined as below: if points *P*, *Q, R* ∈ *E_p_* (*a*,*b*) are on one line, then *P* + *Q* + *R* = *O* (*O* is an infinite point). Given an integer *s* ∈ Z_p_^*^ and a point *P* ∈ *E_p_*(*a*,*b*), the multiplication operation *s* ⋅ *P* over *E_p_*(*a*,*b*) is defined as *P* + *P* + … + *P* in *s* times. If *P* is symmetrical with *P'* on the *X* axis, then *P* + *P'* = *O*. Furthermore, point *P* is a base point with an order *n* if and only if *n* ⋅ *P =* *O*.

### 2.3. ECDLP and CDHP

Every cryptosystem has its own challenges to be resolved, for instance integer factorization in RSA. The most important challenge in ECC is the Elliptic Curve Discrete Logarithm Problem (ECDLP). ECDLP can also lead to several other complexities, such as the Computational Diffie-Hellman Problem (CDLP) or the Elliptic Curve Factorization Problem (ECFP).

**Definition** **1.** Elliptic Curve Discrete Logarithm Problem (ECDLP):

Given two points P and Q over E_p_ (a,b), it is difficult to find an integer s ∈ Z_p_^*^ such that Q = s ⋅ P.

**Definition** **2.** Computational Diffie-Hellman (CDLP):

Given three points P, a ⋅ P and b ⋅ P over E_p_ (a,b), it is difficult to compute the result a ⋅ b ⋅ P.

### 2.4. System Model

Our proposed scheme encompasses four different components such as the cloud server, user, Key Generating Centre (KGC) and auditor.

**Figure 2:**
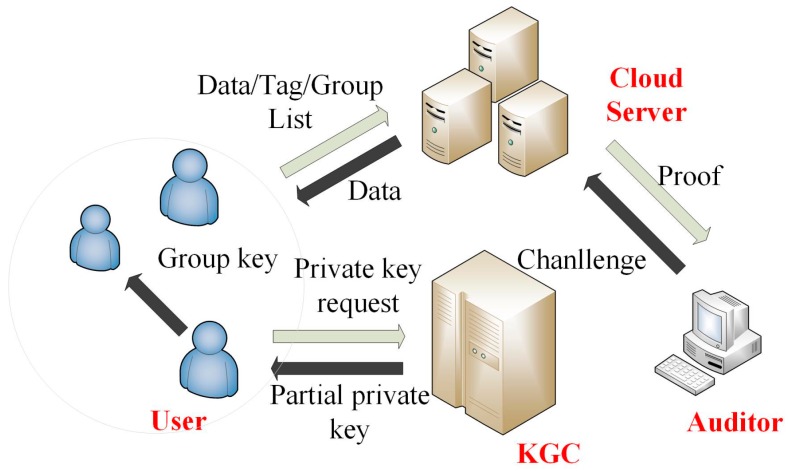
The network model of our proposed public auditing scheme. KGC, Key Generation Centre.

1. Cloud server: The cloud server in our scheme is a semi-trusted entity; the user will upload medical data to the cloud server for storage. Assuming that the cloud server is not fully trusted, user sensitive data stored in the cloud are vulnerable for unauthorized accesses. The cloud server can facilitate the necessary computing power and storage capacity for the WBSNs devices.
2. Key generating centre: KGC is used to generate public parameters for the system and to generate partial public/private keys for users. KGC is a trusted entity.
3. Auditor: The auditor is semi-trusted third party and undertakes the task of data integrity checking. When the user needs to check the integrity of the stored data, the user will request this service through the auditor, and the cloud server runs an interactive algorithm with the auditor to achieve the goal of integrity checking. In this process, the user’s data would not be obtained by the auditor.
4. User: In our scheme, the user is a cloud-assisted WBSN service user. Users use the sensor devices to obtain their own physiological information and to upload the collected physiological information to the cloud server after generating the tag (a signature on the message that is used for checking the integrity of the data) on the collected data and form a historical archive. Users and other group users (such as their community doctors) form a group to share the data stored on the cloud server. Because physiological information is individual private information, users would like to keep their information confidential and do not want any other parties to obtain their data, except the authorized group members. The relationship among these entities is shown in Figure 2.

### 2.5. Security Requirements

*(1)*  Public verifiability: The stored data on the cloud server should be publicly verified by the third party auditor.
*(2)*  Privacy protecting: The uploaded data should not be accessed by the cloud server or auditor even while uploading or auditing.
*(3)*  Multi-user model: Different users can form a group to share the data stored in the cloud server. No other entities except the legal group users can obtain the data stored on the cloud.
*(4)*  Revocability: When users in the group perform illegal operations, the illegal user should be removed from the group by the group manager.
*(5)*  Forward security: In order to ensure forward security, when the illegal user is revoked, the group key should be updated.

## 3. Proposed Scheme

There are fourteen polynomial time algorithms in our proposed schemes, including **Setup**, **PartialPrivateKeyExtract**, **SetSecretValue**, **SetPublicKey**, **SetPrivateKey**, **OrgnaizeGroup**, **JoinGroup**, **Encryption**, **TagGen**, **ProofGen**, **ProofVerify**, **GroupMemberAccessData**, **Decryption** and **Revocation**. These algorithms are mainly divided into four categories: key generating (**Setup**, **PartialPrivateKeyExtract**, **SetSecretValue**, **SetPublicKey**, **SetPrivateKey**), access controlling (**Encryption**, **GroupMemberAccessData**, **Decryption**), group managing (**JoinGroup**, **Revocation**) and auditing (**TagGen**, **ProofGen**, **ProofVerify**). With the algorithm parts of key generating, the KGC can publish the system parameters for system running and generating keys for users including private key, public key and partial public key; in access control parts, three algorithms are provided to prevent the illegal users from accessing data. In our scheme, a group member list that is stored in the cloud server will be used to check if the data user is a legal member in the group. Besides, the legal group members can get a shared group key to decrypt the stored data and without the group key, the illegal users and revoked users cannot access the group sharing data. There are two algorithms in the group managing part: **JoinGroup** and **Revocation**. New users can apply to join the group and get the group key, and the applicant’s identity will be added to the group list, which is maintained in the cloud; if some group members do illegal operation, such as malicious data modification, the group manager will use the **Revocation** algorithm to revoke the malicious group member. The last part of the algorithms used to audit includes of three algorithms: **TagGen**, **ProofGen**, **ProofVerify**. On the user side, before the user wants to upload the data to the cloud, he/she needs to generate the tag on the message for the use of integrity checking; when the user wants to check if the data stored in the cloud are well-kept, he/she can request the auditing service of the auditor; the auditor will generate a challenge to the cloud; and the cloud will compute a proof with the messages to be checked and the corresponding tags (**ProofGen** algorithm). After getting the proof sent from the cloud, the auditor can check if these data are well-kept with the algorithm **ProofVerify**. A brief flowchart can be seen in Figure 2, and Table 1 shows the notations will be used next. The details of each algorithm are described below:

**Setup**: In this phase, the KGC will generate and publish a set of parameters as Algorithm 1 shown below; the other components can get these published parameters. These parameters will be used as the input of the other algorithms and include two cyclic groups, three hash functions and one element generated from the group. The KGC’s public key and master key are also generated in this phase. The master key should be kept by the KGC secretly.
**Algorithm 1. Setup.****Input**: a security parameter *l*
**Output**: the system parameters (*q, G*_1_,*G*_2_, *P*, *h*_1,_
*h*_2,_
*H*, *e*, *Q*, *Q_KGC_*) 1) The KGC chooses a large prime number *q* > 2*^l^*. 2) Chooses an additive group < *G*_1_, +>, a multiplicative group < *G*_2_, >, a generator *P* of *G*_1_, a bilinear paring *e*：*G*_1_ × *G*_1_ → *G*_2_, a point *Q* on *G*_1_. 3) Chooses three hash functions *h*_1_: {{0,1}^*^, *G*_1_} → Z_q_^*^, *h*_2_: {{0,1}^*^, {0,1}^*^,*G*_1_, *G*_1_} → Z_q_^*^, *H*: {0,1}^*^ → *G*_1_. 4) Choose a random number *s_KGC_* ∈ Z_q_^*^ as the master key and generates *Q_KGC_* = *s_KGC_* · *P* as public key. **Returns** (*q, G*_1_, *G*_2_, *P*, *h*_1_, *h*_2_, *H*, *e*, *Q*, *Q_KGC_*).

**PartialPrivateKeyExtract**: Before the user joins the system, he/she needs to apply for the public and private key for him/herself. For the reason that our scheme is based on the certificateless cryptography, the key is separated into two parts. Therefore, if a new user wants to join the system, he/she sends the identity *ID_U_* to KGC for partial key extracting. Upon receiving the user request (the user’s identity *ID_U_*), the KGC runs Algorithm 2 for the requesting user to generate the partial key {*PK_U_*_,1_, *SK_U_*_,1_}. After that, the KGC sends the computed partial key {*PK_U_*_,1_, *SK_U_*_,1_} to the user secretly.
**Algorithm 2. PartialPrivateKeyExtract.****Input**: the user’s identity *ID_U_*
**Output**: the partial key of {*PK_U_*_,1_, *SK_U_*_,1_} 1) The KGC generates a random number *t_U_* ∈ Z_q_^*^. 2) Computes *PK_U_*_,1_ = *t_U_* · *P*. 3) Computes *v_U_* = *h*_1_(*ID_U_*, *PK_U_*_,1_). 4) Computes *SK_U_*_,1_ = *t_U_* + *s_KGC_* · *v_U_* mod *q*. **Returns** {*PK_U_*_,1_, *SK_U_*_,1_}.

**SetSecretValue**: After getting the partial key generated from KGC, the user needs to choose another part of key by him/herself based on the following steps: the user generates a random number *x_U_*∈Z_q_^*^ as his/her secret value. The user sets *SK_U_*_,2_ = *x_U_* and keeps *x_U_* secretly.

**SetPublicKey**: After choosing the secret key by him/herself, he/she computes the corresponding public key with the following steps: user computes *PK_U_*_,2_ = *x_U_* · *P* and sets *PK_U_* = {*PK_U_*_,1_, *PK_U_*_,2_} as his/her public key.

**SetPrivateKey**: User sets *SK_U_* = {*SK_U_*_,1_, *SK_U_*_,2_} as his/her private key. After this stage, all of the setup work of the system has been finished.

**OrgnaizeGroup**: If a user wants to organize a group *ID_G_* (ID*_G_* is the group identity), he/she chooses a random number *x_g_* ∈ Z_q_^*^ as the group encryption key. The group key will be distributed to the legal group members to share the data and prevent the illegal group members from accessing the data.

**Encryption**: Before the data owner with identity *ID_O_* wants to upload the data file *F* to the cloud server, the data owner needs to encrypt *F* with group encryption key *x_g_* as *F ' = F +* (*c*_2_ · *x_g_* · *P*)*_x_*, *R*_2_ = *c*_2_ ·*P* (*c*_2_ is a random integer). The reason we encrypt the data file *F* here is to prevent the illegal user from accessing the data. Besides, for the reason that the data used in the WBSN environment are sensitive physiological data, it is not secure to store these data with plaintext in the cloud because the cloud server is also not trusted and curious about the user’s data.

**TagGen**: After the encryption phase has finished, the encrypted data also have to be tagged for the purpose of integrity checking later. Firstly, the encrypted data file *F'* should be divided into *n* message blocks {*m*_1_, *m*_2_, ..., *m_n_*}. Then, the data owner with identity *ID_O_*, private key *SK_O_* = {*SK_O_*_,1_, *SK_O_*_,2_} and public key *PK_O_* = {*PK_O_*_,1_, *PK_O_*_,2_} runs Algorithm 3 to generate an integrity checking tag (a signature of the message that is used to check the integrity of the data) for every *m_i_* through the following steps, where *I* ∈ {1,2, ..., *n*}. Then, the data owner sends {*m_i_*, *ID_G_*, *id_i_*, *S_i,_ R*_2_} to the cloud server, where *id_i_* is the unique identity of *m_i_*. The cloud server will keep the data {*m_i_*, *ID_G_*, *id_i_*, *S_i,_ R*_2_}, and the legal group user can access the data after verification.
**Algorithm 3. TagGen.****Input**: *SK_O_*, *PK_O_*, *m_i_*, *id_i_*, *ID_G_*, *ID_O_*, *Q_KGC_*, *Q*
**Output**: the tag *S_i_*
1) The data owner computes *v_O_* = *h*_2_ (*ID_G_*, *ID_O_*, *PK_O_*, *Q_KGC_*). 2) Computes *S_i_* = (*SK_O,_*_1_ + *v_O_* · *SK_O,_*_2_) · (*H*(*id_i_*) + *m _i_* · *Q*). **Returns**
*S_i_*.

**ProofGen**: If the user (any legal group members) wants to check if the data stored in the cloud are well-kept, he/she can request the auditing service of the auditor; then, the auditor runs an interactive algorithm with the cloud server to check the integrity of the encrypted data file *F'*. Firstly, the auditor needs to generate an auditing challenge with Algorithm 4.
**Algorithm 4. Challenge.****Input**: NULL **Output**: the challenge {(*i_j_*,*r_j_*)}*_j_*_∈_*_S_*
1) The auditor generates a random subset *S* = {*i*_1_, *i*_2_,..., *i_c_*} from the set Z_n_^*^(1,2,..., *n*) and any two elements are not equal. 2) For every element *i_j_* ∈ *S*, the auditor generates a random number *r_j_* ∈ Z_q_^*^. **Returns** {(*i_j_*,*r_j_*)}*_j_*_∈_*_S_*.

After the auditor has generated the challenge messages, the auditor sends the challenge {(*i_j_*,*r_j_*)}*_j_*_∈_*_S_*_,_ to the cloud server. Upon receiving the auditing challenge {(*i_j_*,*r_j_*)}*_j_*_∈_*_S_*_,_ the cloud server executes Algorithm 5 to generate a proof for the auditor.
**Algorithm 5. ProofGen.****Input**: $m_{i_{j}}$, $S_{i_{j}}$, {(*i_j_*,*r_j_*)}*_j_*_∈_*_S_*
**Output**: the proof {*m_Pro_*, *S_Pro_*} 1) The cloud server computes $S_{\mathrm{Pro}}=\sum_{j=1}^{c}r_{j}\cdot S_{i_{j}}$. 2) Computes $m_{\mathrm{Pro}}=\sum_{j=1}^{c}r_{j}\cdot m_{i_{j}}$ mod *q*. **Returns** {*m_Pro_*, *S_Pro_*}.

After the cloud server has generated the proof, the cloud server sends the proof {*m_Pro_*, *S_Pro_*} to the auditor.

**ProofVerify**: Upon receiving the proof {*m_Pro_*, *S_Pro_*} sent from the cloud server, the auditor runs Algorithm 6 to check the integrity of the data stored in the cloud with {*m_Pro_*, *S_Pro_*}. The algorithm will return a Boolean value. If the value is “TRUE”, the result means that the data stored in the cloud are well kept; if the returning value is “FALSE”, it means that the data file has been damaged.
**Algorithm 6. ProofVerify.****Input**: {*m_Pro_*, *S_Pro_*}, {(*i_j_*,*r_j_*)}*_j_*_∈_*_S_*, *ID_O_*, *PK_O_*, *Q*, *Q_KGC_*
**Output**: TRUE or FALSE 1) The auditor computes *v*_1_ = *h*_1_ (*ID_O_*, *PK_O_*_,1_). 2) Computes *v*_2_ = *h*_2_ (*ID_O_*, *ID_G_, PK_O_*, *Q_KGC_*). 3) Then third-party auditor can check the integrity of the stored data by verifying whether equation *e*(*S_Pro_*, *P*) = *e*(*m_Pro_* · *Q* + $\sum_{j=1}^{c}$*r_j_* · *H*($id_{i_{j}}$), *PK_O_*_,1_ + *v*_1_ · *Q_KGC_* + *v*_2_ · *PK_O_*_,2_) holds. If holds, **returns** TRUE, Else, **returns** FALSE.

**GroupMemberAccessData**: When the group member with identity *ID_B_* needs to access data, he/she sends (*ID_B_*, *ID_G_*) to the cloud server. The cloud server checks if *ID_B_* is a valid member in this group with group member list *L_G_*. If not, the cloud server terminates the operation; else, the *ID_B_* is a legal member in this group. Therefore, the cloud server can send the encrypted data (*F', R*_2_) to the user.

**Decryption**: After getting the encrypted data file (*F'*, *R*_2_) from the cloud server. The group member *ID_B_* can decrypt the encrypted data file *F* with group secret key *x_g_ as*: *F =* *F'*− (*x_g_* · *R*_2_)*_x_*. and get the group shared data.

**Revocation**: In our scheme, the group members are self-organized. The group organizer has the right to revoke the members who perform illegal actions, such as illegal data modification. When the group organizer wants to revoke or update group members, he/she needs to do the following steps:

1. Update the group members list *L_G_* (the illegal user’s identity information is removed from the *L_G_*). Then, the group organizer sends the updated group members list *L_G_'* to the cloud server.
2. The organizer chooses a new random number *x_g_'* ∈ Z_q_^*^ as a new group encryption key and distributes the key to all other legal group members. The new group key distributing method is same as that in the algorithm **JoinGroup**.

At this step, all of the algorithms in our proposed schemes have been introduced.

## 4. The Proven Security of Our Proposed Scheme

In this section, we will prove that our scheme is secure against public key replacement attack in the random oracle model. In Section 4.1, we introduce the basic concept of proven security firstly; the security model of our scheme is introduced in Section 4.2; lastly, the Section 4.3 is our proof process.

### 4.1. The Basic Concept of Proven Security

As one kind of axiomatic research method, the proven security theory is the most widely-used analysing tool to analyse the security of the cryptographic protocols. The basic idea of provable security is to deduce the proving scheme to a known secure scheme or “extremely primitive” (such as the discrete logarithm problem) and produce a paradox. The process of this proof is actually using the apagoge mathematical proof: assume that the attacker can construct a polynomial time algorithm to solve our security problem, then the polynomial time algorithm can solve the difficulties of the primitive, which is a known secure problem (difficult to solve with the existing technology), then we know that our assumption is wrong. At this stage, the process of the proof is mainly based on the random oracle model.

**Figure 3:**
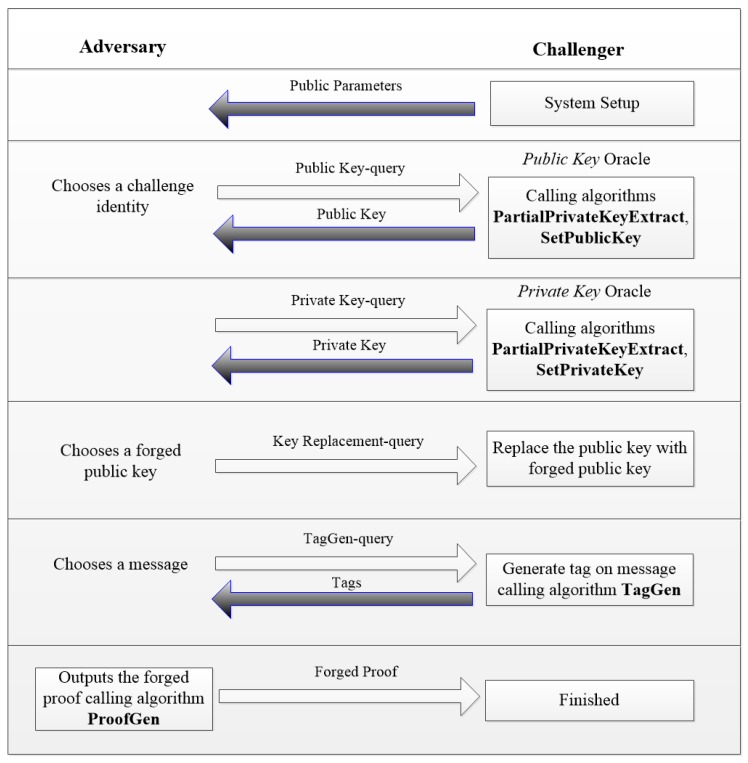
The flowchart of the attacking games in our security proof.

Generally, there are several steps in the proof process:

(1) The formal definition of cryptographic protocols: the original scheme should be abstracted into a conversion scheme, which can deduced a difficult problem (“extremely primitive”).
(2) Set the security goals to be achieved. As in a signature scheme, we want to see that our scheme could defend against the CMA (Chosen Message Attack). In our scheme, we want to see that our scheme could defend against the public key replacement attack [41,43].
(3) The establishment of the security model: The establishment of security model is mainly to determine the attacker’s attacking purpose and attacking ability. For example, in the digital signature protocol, the adversary’s target is to obtain the ability to forge the signature of any messages or to obtain the private key of the signer. Attacking ability is a description of the attacking steps in order to achieve the goal. As in the digital signature scheme, the adversary can choose any message other than the challenge identity to ask the signature of the signature machine.
(4) Formal proof: This process is the core technology of provable security theory. Proven by formal methods to establish an attacking game between analogue rival and challenger to inverse the attacking process, then it converts to solving difficult problems, such as the large prime factorization problem, the discrete logarithm problem in a finite field, etc., thus completing the proof. Many existing cryptographic schemes have been proven secure in the random oracle model [30,33,34,41,43]. The flowchart of the attacking games in our security proof is shown in Figure 3.

Based on the basic proof idea above, our proof process sets up an attacking game between an attacker and a challenger. The challenger initiates several oracle machines, and the attacker can query the challenger. With the query responses, the attacker is able to launch a replacement key attack, which means that the attacker can replace the users’ partial public key; however, the generated signature with this replaced key could pass the verifying phase [41,43]. We will prove that below: if the attacker can successfully replace the user’s public key (partial public key) and pass the verification phase (**ProofVerify** algorithm), then the attacker can use the attacker's attacking algorithm to solve the known secure “extremely primitive”- CDH problem with an instance of (*P*, *Q*_1_ = *a* · *P*, *Q*_2_ = *b* · *P*).

### 4.2. Security Model

In our security assumption, an attacker can launch a key replacement attack. In the initial stages, the challenger runs the algorithm **Setup** to generate the system parameters and extract the user’s partial-public and partial-private key. The challenger then returns the public key of the user and system parameters to the attacker; the attacker is able to replace the public key of the user and required for the oracle machines controlled by the challenger below (Figure 3 shows the flowchart of the attacking games in our security proof):

**h_1_-query**: upon receiving a query with the user’s identity and the corresponding partial public key (*ID_U_*, *PK_U_*_,1_), the challenger returns the hash value *v_U_* to the adversary.

**h_2_-query**: upon receiving a query with user’s identity, the group identity, the public key of data owner and the KGC’s public key (*ID_O_*, *ID_G_, PK_O_*, *Q_KGC_*), the challenger returns the hash value *v_O_* to the adversary.

**H-query**: upon receiving a query with the message block’s identity *id_i_*, the challenger returns the hash value *y_i_* · *Q*_2_ *− m_i_* · *Q* to the adversary.

**PublicKey-query**: upon receiving a query with user’s identity *ID_O_*, the challenger runs the algorithms **PartialPrivateKeyExtract** and **SetPublicKey** to generate the user’s public key {*PK_U_*_,1_,*PK_U_*_,2_} and returns the value {*PK_U_*_,1_,*PK_U_*_,2_} to the adversary.

**PrivateKey-query**: upon receiving a query with user’s identity *ID_O_*, the challenger runs the algorithms **PartialPrivateKeyExtract** and **SetSecretValue** to generate the user’s private key {*SK_U_*_,1_, *SK_U_*_,2_} and returns the value {*SK_U_*_,1_, *SK_U_*_,2_} to the adversary.

**Keyreplacement**: upon receiving the request with forged public key generated by the attacker and the attacking target’s identity (*ID_U_*, *PK_U_*_,1_^’^, *PK_U_*_,2_^’^), the challenger replaces the (*PK_U_*_,1_, *PK_U_*_,2_) with (*PK_U_*_,1_^'^, *PK_U_*_,2_^'^).

**TagGen**: upon receiving a query with a user’s identity *ID_U_* and data *m_i_* with identity *id_i_*, the challenger returns the integrity checking tag with the **TagGen** algorithm.

### 4.3. Security Proof

**Lemma** **1.** Our proposal is secure against the public key replace attack with the assumption that the CDH problem is hard.

**Proof:**  Suppose that the attacker can successfully replace the user’s public key (partial public key) and pass the verification phase (**ProofVerify** algorithm), then we could construct a challenger using attacker’s attacking algorithm as the sub-routine to solve the CDH problem with a non-negligible probability: with an instance of (*P*, *Q*_1_ = *a* · *P*, *Q*_2_ = *b* · *P*), the challenger sets *Q_KGC_*←*Q*_1_ and publishes the system parameters (*q, G*_1_, *G*_2_, *P*, *h*_1_, *h*_2_, *H*, *e*, *Q*, *Q_KGC_*) to the attacker. Then, the challenger chooses a challenging identity *ID_U_* and answers the queries from the adversary below:

**h_1_-query**: the challenger maintains a hash list *L_h_*_1_ (*ID_U_*, *PK_U_*_,1,_ *v_U_*) and *L_h_*_1_ is initialized to empty. If an adversary submits a request with (*ID_U_*, *PK_U_*_,1_）, the challenger checks whether tuple exists in *L_h_*_1_. If it exists, the challenger returns the value *v_U_* to the adversary; otherwise, the challenger generates a random number *v_U_* ∈Z_q_^*^ and returns *v_U_* to the adversary.

**h_2_-query**: the challenger maintains a hash list *L_h_*_2_(*ID_O_*, *ID_G_, PK_O_*, *Q_KGC_, v_O_*), and *L_h_*_2_ is initialized to empty. If an adversary submits a request with (*ID_O_*, *ID_G_, PK_O_*, *Q_KGC_*）, the challenger checks whether tuple exists in *L_h_*_2._ If existing, the challenger returns the value *v_O_* to adversary; otherwise, the challenger generates a random number *v_O_* ∈ Z_q_^*^ and returns *v_O_* to the adversary.

**H-query**: the challenger maintains a hash list *L_H_*(*id_i_*, *y_i_*, *Y_i_*) and initialized as empty. If an adversary submits a request with *id_i_*, the challenger checks whether the tuple exists in *L_H_*. If existing, the challenger returns the value *Y_i_ − m_i_*·*Q* to the adversary; otherwise, the challenger generates a random number *y_i_* ∈ Z_q_^*^, returns *y_i_*·*Q*_2_ *− m_i_*·*Q* to the adversary.

**PublicKey-query**: the challenger maintains a list *L_PK_*(*ID_U_*, *PK_U_*_,1_, *PK_U_*_,2_, *SK_U_*_,1_, *SK_U_*_,2_) and initialized as empty. If an adversary submits a request with *id_i_*, the challenger checks whether the tuple exists in *L_PK_*. If existing, the challenger returns {*PK_U_*_,1_, *PK_U_*_,2_} to the adversary; otherwise, the challenger generates random numbers *SK_U_*_,1_, *SK_U_*_,2,_ *x_U_* ∈ Z_q_^*^ and returns *PK_U_*_,2_ = *SK_U_*_,2_ · *P* and *PK_U_*_,1_ = *SK_U_*_,1_ · *P − x_U_* · *Q_KGC_* to the adversary.

**PrivateKey-query**: the challenger maintains a list *L_SK_*(*ID_U_*, *SK_U_*_,1_, *SK_U_*_,2_) and initialized as empty. If an adversary submits a request with *ID_U_*, the challenger checks whether the tuple exists in *L_SK_*. If existing, the challenger returns {*SK_U_*_,1_, *SK_U_*_,2_} to the adversary; otherwise, the challenger aborts the game.

**KeyReplacement**: upon receiving the request with (*ID_U_*, *PK_U_*_,1_^'^, *PK_U_*_,2_^'^), the challenger checks if *ID_U_* exists in *L_PK_.* If it exists, the challenger replaces (*PK_U_*_,1_, *PK_U_*_,2_) with (*PK_U_*_,1_^'^, *PK_U_*_,2_^'^).

**TagGen**: upon receiving a query with a user’s identity *ID_U_* and data *m_i_* with identity *id_i_*, the challenger makes an H-query with *id_i_* and gets tuple {*id_i_*, *y_i_*, *Y_i_*}. Then, the challenger will compute (*SK_U,_*_1_ + *v_O_* · *SK_U,_*_2_) · (*Y_i_* + *m_i_* · *Q*) and returns the result to the adversary.

**ProofGen**: finally, the adversary outputs a proof {*m_Pro_^'^*, *S_Pro_^'^*} of a subset {*i*_1_, *i*_2_,..., *i_c_*} corresponding to the owner’s identity *ID_O_* as his/her forgery. ☐

Then, the challenger can get equation:

(1) $$\begin{array}{c} e(S_{\mathrm{Pro}},P)\\ =e(\sum_{j=1}^{c}r_{j}\cdot S_{i_{j}},P)\\ =e(m_{\mathrm{Pro}}\cdot Q+\sum_{j=1}^{c}r_{j}\cdot H(id_{i_{j}}),{PK}_{U,1}+v_{o}\cdot {PK}_{U,2}+v_{U}\cdot Q_{\mathrm{KGC}})\\ =e(m_{\mathrm{Pro}}\cdot Q+\sum_{j=1}^{c}r_{j}\cdot H(id_{i_{j}}),{PK}_{U,1}+v_{o}\cdot {PK}_{U,2})\times e(m_{\mathrm{Pro}}\cdot Q\sum_{j=1}^{c}r_{j}\cdot H(id_{i_{j}}),\;v_{U}\cdot Q_{\mathrm{KGC}})\\ =e(\sum_{j=1}^{c}r_{j}\cdot Y_{i_{j}},{PK}_{U,1}+v_{o}\cdot {PK}_{U,2})\times e(\sum_{j=1}^{c}r_{j}\cdot Y_{i_{j}},v_{U}\cdot Q_{\mathrm{KGC}}) \end{array}$$

For the reason that the public key was successfully replaced, the forging tag could also pass the verifying phase, so we get the equations below, as well:

(2) $$\begin{array}{c} e({S_{\mathrm{Pro}}}^{\prime },P)\\ =e(\sum_{j=1}^{c}r_{j}\cdot S_{i_{j}},P)\\ =e(m_{\mathrm{Pro}}\cdot Q+\sum_{j=1}^{c}r_{j}\cdot H(id_{i_{j}}),{PK}_{U,1}+v_{o}\cdot {PK}_{U,2}+v_{U}\cdot Q_{\mathrm{KGC}})\\ =e(\sum_{j=1}^{c}r_{j}\cdot Y_{i_{j}},{PK}_{U,1}+v_{o}\cdot {PK}_{U,2})\times e(\sum_{j=1}^{c}r_{j}\cdot Y_{i_{j}},{v_{U}}^{\prime }\cdot Q_{\mathrm{KGC}}) \end{array}.$$

with Equations (1) and (2), we can compute (1)/(2) and get the solution of the CDH instance as: ${a\cdot b\cdot P=(S_{\mathrm{Pro}}-{S_{\mathrm{Pro}}}^{\prime }-(v_{U}-{v_{U}}^{\prime })\cdot \sum_{j=1}^{c}r_{j}\cdot y_{i_{j}}\cdot Q_{\mathrm{KGC}})}^{-1}$

It can be observed that if the adversary can forge a message passing the verifying phase successfully, the CDH problem could be addressed by the challenger. However, the integrity of the CDH problem prevents forging of a message through key replacement attack.

Through the proof above, we could see that our scheme can resist a key replacement attack in the random oracle model.

## 5. Security Analysis

This section describes how our proposal can satisfy the security requirements mentioned previously. Table 2 shows the comparison with similar proposals [41,43].

Public verifiability: Using algorithms **ProofGen** and **ProofVerify**, the cloud server can prove the integrity of the stored data to a third part auditor. In our scheme, the cloud server is a semi-trusted party, which means that the cloud server will comply with the communication protocol fully. Based on this assumption, public verifiability is achieved.

Multi-user Model: In our scheme, the group key is encrypted with the public key of each group member and is transmitted to all members in the group. The group membership list is sent to the cloud storage server. Legal group members will be able to obtain group sharing data from the cloud server and decrypt the cipher text with a group key.

Revocability: When members in the group perform illegal operations, the illegal users should be removed from the group by the group manager. The group manager updates the list of members, and this new list is sent to the cloud server; the group manager generates a new group key and sends it to other legitimate users (except of the revoked user).

Forward security: In our scheme, the group key will be updated when group members are updated. The new group key will be distributed to legal users.

Privacy protecting: Privacy protection in our scheme can be proven based on the two following perspectives. (1) Before the data file is divided into blocks, it is encrypted with a group key, so the message uploaded to the cloud server is encrypted. Even if the cloud server reorganizes the data block, the cloud server can only be able to obtain the cipher text. The encryption key is not available for the cloud server. (2). In the process of public auditing, the third party auditor can only obtain data {*m_Pro_*, *S_Pro_*} from the cloud server. However, with $S_{\mathrm{Pro}}=\sum_{j=1}^{c}r_{j}\cdot S_{i_{j}}$ and $m_{\mathrm{Pro}}=\sum_{j=1}^{c}r_{j}\cdot m_{i_{j}}$ mod *q*, the auditor cannot access any contents of the stored data.

## 6. Experimental Evaluation

### 6.1. Experimental Parameters

In this section, we implement our scheme on a machine with Intel Core i5-3337U CPU (1.8G Hz clock speed) and 4GB RAM. The running operating system is Windows 8 and the IDE with Eclipse luna. The testing number of data blocks is set as [0,1000], and every block size is 5 KB; the number of requesting users is set as [0,100]. Recently, many cryptographic libraries have been implemented, such as MIRACL (Multiprecision Integer and Rational Arithmetic C/C++ Library), JPBC (Java Pairing Based Cryptography) [49], and so on. Here, we implement our proposal with JPBC in JAVA language. In some algorithms, such as **ProofGen**, **TagGen**, **Encryption** and **Decryption**, the algorithm efficiency is related to the number of processed data numbers, but in some algorithms, such as **PartialPrivateKeyExtract** or **JoinGroup**, the efficiency will be affected by the number of requested users. Therefore, with these experiments, we want to show the relationship between the security computation cost of each algorithm with the number of data blocks or requesting users.

In our experiment, the Type A pairings in the JBPC library are used, which is constructed on the curve *y*^2^ = *x*^3^ + *x* over the field *F_q_* for some prime *q* *=* 3 mod 4. Both *G*_1_ and *G*_2_ are the groups constructed by the points selected from the elliptic curve.

*E*(*F_q_*), so this pairing is symmetric. It turns out #*E*(*F_q_*) = *q* + 1 and #*E*(*F_q_*_2_) = (*q* + 1)^2^. Thus, the embedding degree *k* is 2, and hence, *G_T_* is a subgroup of *F_q_*_2_. The order *r* is some prime factor of *q* + 1. Write *q* + 1 = *r* * *h*, and *h* is some number. For efficiency, *r* is picked to be a Solinas prime, that is *r* has the form of 2*^a^* + ( − 2*^b^*) + (−1) for some integers 0 < *b* < *a*. Furthermore, we choose *q* = −1 mod 12 so *F_q_*_2_ can be implemented as *F_q_*[*i*] (where *i* = sqrt(−1)), and since *q* = −1 mod 3, cube roots in *F_q_* are easy to compute.

### 6.2. Experimental Analysis

**Figure 4:**
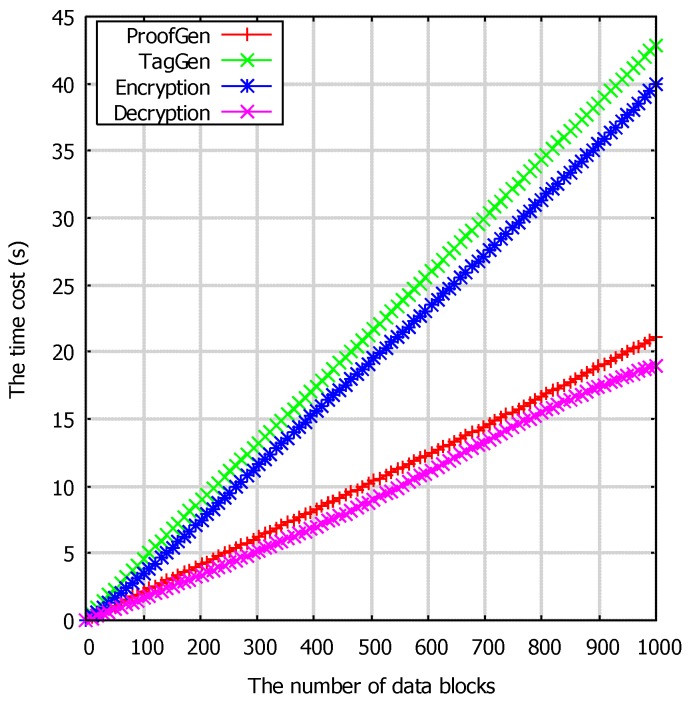
The time cost of the algorithm **ProofGen**, **TagGen**, **Encryption** and **Decryption** with regard to the numbers of data blocks in seconds.

We tested our scheme in each phase, and the running time of each algorithm is obtained. In Figure 4, we listed the time cost of the cryptographic operation in different numbers of messages including of **ProofGen**, **TagGen**, **Encryption** and **Decryption**. For the reason that in pairing-based cryptographic schemes, the point multiplication operation, hash to point and bilinear pairing are the main computational expansive operations, so the time cost of each algorithm mainly depends on the number of point multiplication operations. For example, in the **TagGen** algorithm, the cryptographic is almost 2*n* PM + *n* H, where the PM means point multiplication and H means hash to point operation, but in the **ProofGen** phase, only a 1 PM cryptographic operation is needed, so the **TagGen** algorithm needs almost double that in the **ProofGen** phase.

**Figure 5:**
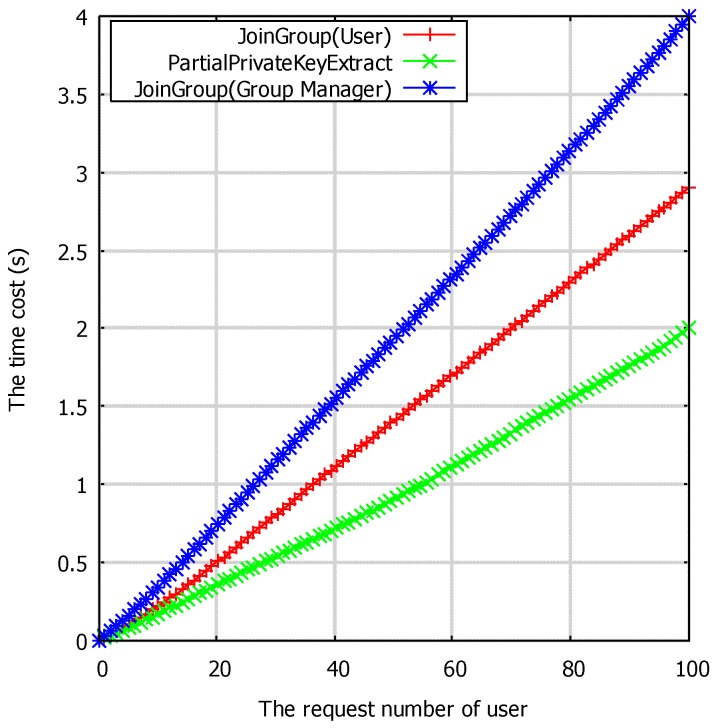
The time cost of the algorithms **PartialPrivateKeyExtract** and **JoinGroup** with regard to the requesting number of users in seconds.

Figure 5 shows the running time of **PartialPrivateKeyExtract** and **JoinGroup** in different numbers of requesting users. To generate the partial key for the user, the KGC needs to do 1 PM + 1 h cryptographic operations. In the **JoinGroup** phase, we separate the cryptographic operation into two aspects: on one side, the group manger needs to do about 2 PM to distribute the secret group key to the applicant; on another side, the applicant needs to do about 1 PM to get the secret group key. A detailed comparison of the cryptographic operations of different algorithms can be checked in Table 3. In Table 3, the PM means point multiplication operation, h means one hash function, H means the hash to point operation and E means the bilinear pairing. The variable *n* in the **TagGen**, **ProofGen** and **ProofVerify** algorithms means the data blocks to generate the tag or to verify.

**Figure 6:**
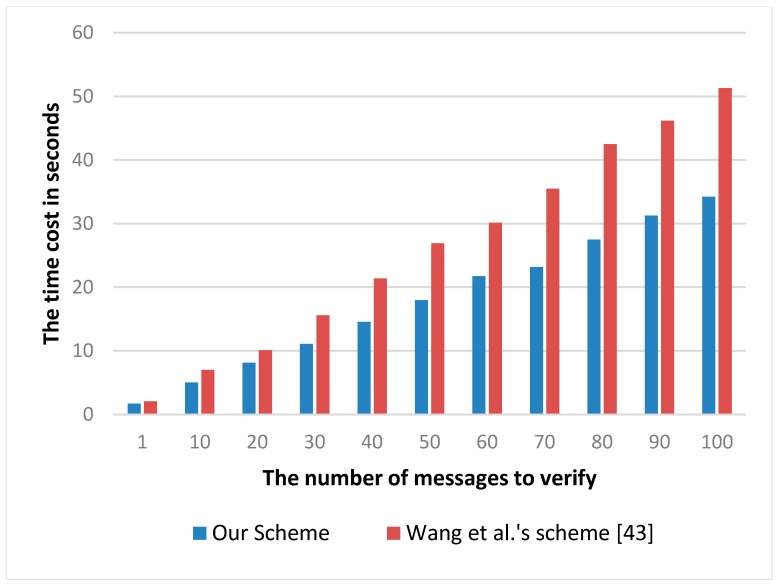
The time cost of the **ProofVerify** algorithm on the auditor sider with regard to the number of blocks in seconds.

Figure 6 compares the efficiencies of our scheme with Wang et al.’s scheme [43]. It can be observed that for **ProofVerify**, our scheme achieves better efficiency performance compared to Wang et al.’s scheme [43]. In Wang et al.’s scheme [43], three paring operations are needed, but only two pairing operations are needed in our scheme. Compared with the other cryptographic operations such as symmetrical encryption or the hash function, pairing requires more computational cost (about several dozen times). Therefore, from Figure 6, we can see that compared with Wang et al.’s scheme, it is clear that our scheme improved the proof verifying efficiency of auditor to nearly 30%.

## 7. Conclusions

This paper proposes a cloud-assisted WBSNs public auditing scheme in a multi-user model with privacy protection. Our scheme not only satisfies the property of third party public auditing, but also supports a multi-user model for effective deployment of WBSN applications in community environments such as community hospitals. With the property of the revocation of illegal users and the group key updating mechanism, the system’s forward security is protected. In our proposed scheme, no other participating entities including the cloud server or the third party auditor can access the content of the uploaded data, which ensures data integrity and user confidentiality. With the property of certificateless encryption, the storage and bandwidth requirements of our proposed scheme are greatly reduced. Moreover, our proposed scheme is more suitable for cloud-assisted WBSN applications and outperforms other existing schemes in the same context.

## Figures and Tables

**Table 1:** The notations used in our scheme.

Symbol	The Meaning of the Symbol
*l*	A security parameter
*q*	A large prime number *q > 2^l^*
*e*	A bilinear pairing *e*: *G*_1_ × *G*_1_ → *G*_2_
*s_KGC_*	The master key
*Q_KGC_*	The public key of KGC
*ID_U_*	The identity of user
*PK_U_*_,2_	The partial public key generated by user self
*PK_U_*_,1_	The partial public key generated by KGC
*SK_U_*_,2_	The partial secret key generated by user self
*SK_U_*_,1_	The partial secret key generated by KGC
*x_g_*	The group shared encryption key
*L_G_*	The group member list
*h*_1_	The hash functions {{0,1}^*^, *G*_1_} → Z_q_^*^
*h*_2_	The hash functions {{0,1}^*^, {0,1}^*^,*G*_1_, *G*_1_} → Z_q_^*^
*H*	The hash functions {0,1}^*^ → *G*_1_

**Table 2:** Comparison of the security properties of the three proposals.

Property	Wang et al.’s Scheme [43]	He et al.’s Scheme [41]	Our Scheme
Public verifiability	√	√	√
Multi-user sharing	×	×	√
Revocability	N/A	N/A	√
Forward security	√	√	√
Privacy protection	×	×	√
Batch authentication	√	√	√
Proven security	√	√	√
Key replacement resistant	×	√	√

**Table 3:** The cryptographic operations in each algorithm.

PartialPrivateKeyExtract	JoinGroup (Group Manager)	JoinGroup (User)	Encryption	TagGen	ProofGen	ProofVerify	Decryption
1 PM + 1 H	2 PM	1 PM	2 PM	2*n* PM + *n* H	*n* PM	2E + (*n* + 3) PM + *n* H	1 PM
